# RNA Binding Proteins as Drivers and Therapeutic Target Candidates in Pancreatic Ductal Adenocarcinoma

**DOI:** 10.3390/ijms21114190

**Published:** 2020-06-11

**Authors:** Markus Glaß, Patrick Michl, Stefan Hüttelmaier

**Affiliations:** 1 Institute of Molecular Medicine, Martin Luther University Halle-Wittenberg, Charles Tanford Protein Center, Kurt-Mothes-Str. 3a, 06120 Halle, Germany; stefan.huettelmaier@medizin.uni-halle.de; 2 Universitätsklinik und Poliklinik für Innere Medizin I, Universitätsklinikum Halle (Saale), Ernst-Grube-Str. 40, 06120 Halle (Saale), Germany; patrick.michl@uk-halle.de

**Keywords:** RNA binding proteins (RBPs), pancreatic ductal adenocarcinoma (PDAC), APOBEC1, IGF2BP1, IGF2BP3, OASL

## Abstract

Pancreatic ductal adenocarcinomas (PDAC) belong to the most frequent and most deadly malignancies in the western world. Mutations in KRAS and TP53 along with some other frequent polymorphisms occur almost universally and are likely to be responsible for tumor initiation. However, these mutations cannot explain the heterogeneity in therapeutic responses observed in PDAC patients, which limits efficiency of current therapeutic strategies. Instead, recent classifications of PDAC tumor samples are based on transcriptomics data and thus include information about epigenetic, transcriptomic, and post-transcriptomic deregulations. RNA binding proteins (RBPs) are important post-transcriptional regulators involved in every aspect of the RNA life cycle and thus considerably influence the transcriptome. In this study, we systematically investigated deregulated expression, prognostic value, and essentiality reported for RBPs in PDAC or PDAC cancer models using publicly available data. We identified 44 RBPs with suggested oncogenic potential. These include various proteins, e.g., IGF2 mRNA binding proteins (IGF2BPs), with reported tumor-promoting roles. We further characterized these RBPs and found common patterns regarding their expression, interaction, and regulation by microRNAs. These analyses suggest four prime candidate oncogenic RBPs with partially validated target potential: APOBEC1, IGF2BP1 and 3, and OASL.

## 1. Introduction

Pancreatic cancer currently is the fourth leading cause of cancer-associated death in Western societies and predicted to become the second leading cause of death by 2030 [1,2]. The vast majority of pancreatic malignancies are pancreatic ductal adenocarcinomas (PDACs), accounting for more than 95% of pancreatic malignancies [3]. PDAC has one of the worst prognoses of any common solid tumors with a 5-year survival rate of around 5–8% [1,4]. The adverse prognosis in most cases is due to diagnosis at advanced disease stages and only up to 20% of patients are candidates for surgical resection in curative intent [5,6]. Most PDACs are associated with somatic mutations, most frequently in the KRAS, TP53, CDKN2A, and SMAD4 genes. Especially, KRAS mutations were found to occur in more than 90% of all tumor samples [3,7]. In transgenic mouse PDAC models, activating mutations in the K-ras gene are typically sufficient for the initiation of tumorigenesis [3]. However, these known and frequent mutations do not allow clinically relevant prognostic classifications, suggesting that the origins of the PDAC heterogeneity may be found at the postgenetic level, since therapy resistance and poor outcome is associated with substantial transcriptome heterogeneity observed in PDAC [6]. At least three PDAC subtype classifications based on transcriptomic data have been proposed. Moffitt et al. [8] described two tumor-specific subtypes obtained via non-negative matrix factorization (NMF) of microarray data; a basal-like subtype, typically associated with worse outcome, and a classical subtype with improved prognosis. Furthermore, this study proposed two classes of stroma subtypes, normal and activated, yielding four molecular PDAC subtypes in total. Collisson et al. [9] suggested three subtypes also generated by NMF of microarray data: classical, quasi-mesenchymal, and exocrine-like. Notably, the classical subtypes proposed by Moffitt et al. and Collison et al. show substantial overlaps in gene expression signatures. Bailey et al. [1] derived four subclasses by applying NMF to RNA-seq and microarray data of pancreatic cancer samples: squamous, pancreatic progenitor, immunogenic, and aberrantly differentiated endocrine exocrine (ADEX). Application of these three classification systems to the RNA expression data provided by The Cancer Genome Atlas (TCGA) research network revealed that classification of the samples as basal-like or classical was independent of tumor purity. Moreover, these subtypes were distinguished by differential regulation of gene expression by microRNAs (miRNAs) and DNA methylation [7]. Juiz et al. reported that the basal-like and classical subtypes can be interconverted by upregulation or downregulation of the transcription factors MET and GATA6, which were proposed to regulate disease super-enhancers, i.e., clusters of transcription factors located in the same region of the genome [6]. According to Juiz et al., PDAC carcinogenesis is initiated by mutations triggering epigenetic deregulation. This was proposed to drive transcriptome alterations that finally manifest in the basal-like or classical PDAC subtype. Gene expression is regulated at various levels including RNA-dependent regulation by short and long noncoding RNAs (ncRNAs) as well as RNA binding proteins (RBPs). While miRNAs as one specific class of ncRNAs have been studied extensively in pancreatic cancer (e.g., [10,11,12]), little is known about the role of RBPs in PDAC progression. Despite the lack of systematic investigation of RBPs in PDAC, ample evidence suggests substantial impact of some RBPs in this malignancy. For example, all three members of the insulin-like growth factor 2 mRNA binding protein family (IGF2BPs) have recently been described as being upregulated and associated with a poor prognosis in pancreatic cancer [13,14,15,16,17]. Furthermore, inactivation of the histone deacetylase SIRT6 was reported to accelerate PDAC progression in mouse models due to upregulation of LIN28B [18]. Consistent with the role of LIN28B in impairing biogenesis of let-7 microRNAs [19], LIN28B upregulation was associated with downregulation of let-7 microRNAs and upregulation of major let-7 targets HMGA2, IGF2BP1, and IGF2BP3. Intriguingly, IGF2BP1, LIN28B, and HMGA2 form a self-promoting network antagonizing the tumor-suppressive actions of the let-7 miRNA family [20]. Other RBPs with suggested tumor-promoting roles in PDAC include the 5’-3’ exonuclease EXO1 [21] and the RBPs HuR (ELAVL1) and PTBP3, which both have been reported to lead to hypoxia-induced chemoresistance in pancreatic cancer cells [22,23].

In the present study, we analyzed publicly available data from high-content studies to determine which RBPs distinguish the classical from the basal-like PDAC subtype and which of these may play tumor-promoting or -suppressing roles in order to identify novel candidate targets for diagnosis and therapy.

## 2. Results

### 2.1. Workflow for the Identification and Characterization of Oncogenic RBPs in PDAC

**Figure 1:**
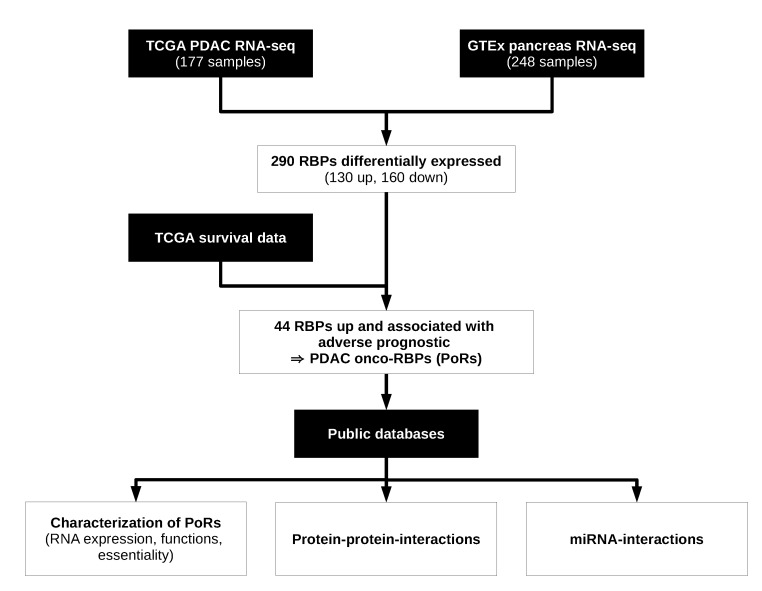
Analysis of pancreatic ductal adenocarcinomas (PDAC) onco-RNA binding proteins (RBPs). Flowchart depicting the analysis pipeline for the *in silico* identification and characterization of RBPs with oncogenic characteristics in PDAC using public data sets. Black boxes represent data sources, white boxes denote analysis steps.

In order to identify RBPs showing oncogenic or tumor suppressive potential in PDAC and its subtypes, we first determined differential gene expression using large-scale transcriptomics data from The Cancer Genome Atlas (TCGA) and the Genotype-Tissue Expression (GTEx) projects [7,24]. Next, we combined the results of this analysis with survival data to evaluate which RBPs deregulated in PDAC were associated with adverse or beneficial prognoses. This led to 44 RBPs that showed an elevated RNA expression in PDAC or at least one of its subtypes and that, in addition, showed adverse prognosis when expressed at a higher level. We termed them PDAC oncogenic RBPs or PoRs. We further examined these 44 RBPs regarding various characteristics and common properties, putative protein–protein interactions among them, and possible miRNA interactions (Figure 1).

### 2.2. Transcriptional Regulation in PDAC

**Figure 2:**
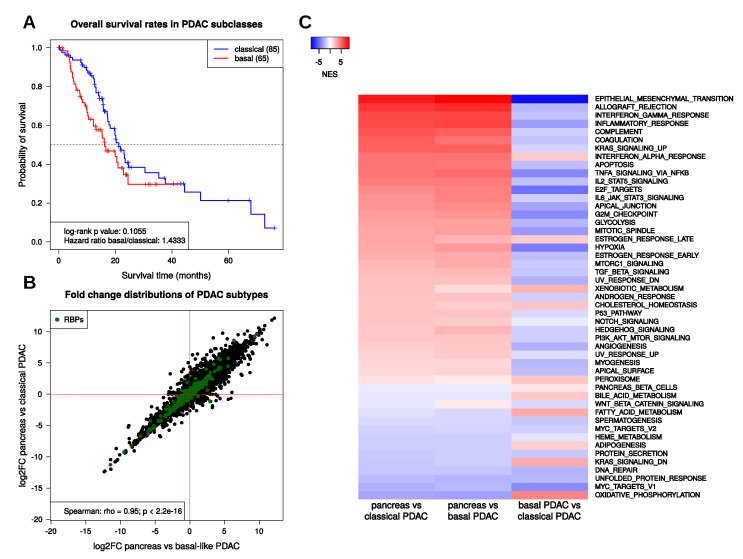
PDAC subtype properties. (**A**) Kaplan–Meier-curves showing overall survival rates for classical (blue) and basal-like (red) PDAC. (**B**) RNA expression changes of protein-coding genes in classical and basal-like PDAC subtypes compared to normal pancreas samples. Green points mark RNA binding proteins (RBPs). (**C**) Heatmap showing normalized enrichment scores (NES) obtained from gene set enrichment analyses (GSEA) of the 50 MSigDB hallmark gene sets using fold changes obtained by the comparisons of pancreas against classical PDAC (first column), pancreas against basal-like PDAC (second column), and basal-like against classical PDAC (third column).

To infer information about deregulation of RBPs on the RNA level, we used PDAC RNA-seq data derived from the TCGA project [7]. However, these data only contain four normal tissue samples. Therefore, we decided to compare the primary tumor samples with pancreas RNA-seq data from the GTEx project, providing transcriptome data of nonmalignant human tissue [24]. We used raw count data from both projects and processed them together to avoid RNA composition biases (see Methods). Altogether, we compared 248 pancreas samples with 177 PDAC tumor samples. Gene set enrichment analyses (GSEA, [25]), using the fold change of expression to rank genes, revealed pancreatic-cancer-related gene sets among the most significantly enriched sets (Supplementary Figure S1A; Supplementary Table S1). In total, 7641 out of 18,797 investigated protein-coding genes were determined as differentially expressed ($FDR<0.05,|log_{2}FC|\geq 1$). Notably, more than twice as many genes were upregulated (5221) than downregulated (2420) in PDAC samples, suggesting a substantial reprogramming and reactivation of the transcriptome silenced in nonmalignant pancreatic tissue (Supplementary Figure S1B). Protein-coding transcripts showing the highest expression values in normal pancreas tissue all encode for digestive enzymes and cofactors. These proteins showed a markedly lower expression in PDAC samples, supporting the observed impairment of exocrine functions of the pancreas (Supplementary Figure S1C). In addition to considering the complete PDAC sample set, gene expression of pancreas tissue was compared to the expression of the two major molecular subtypes of PDAC, basal-like and classical, as well as between both subtypes. The classification of the TCGA tumor samples into these subtypes was obtained from the TCGA Research Network [7]. Of the 177 PDAC tumor samples, 84 matched the classical and 65 matched the basal-like subtype. Survival analysis of the subtype samples confirmed the trend that the basal-like subtype is associated with a lower survival probability (hazard ratio basal/classical = 1.4, log-rank test *p* value = 0.1; cf. Figure 2A). Interestingly, deregulated gene expression determined between pancreatic tissue and PDAC subtypes revealed a high correlation of fold changes observed in classical and basal-like subtype samples (Figure 2B). This suggests that the differences between the two subtypes might not rely on fundamental differences in gene expression but rather implies distinct pronunciation of the expression of specific genes. This was further analyzed by GSEA to determine processes and pathways containing genes particularly deregulated in the two PDAC subtypes compared to normal pancreas and to each other. For this purpose, the 50 MSigDB hallmark gene sets were used. These sets are comprised of genes serving as markers of well-defined biological states or processes [26] (Figure 2C). Overall, normalized enrichment scores (NES) determined by GSEA were similar for the classical and for the basal-like subtype, indicating analogous trends in deregulation of the processes represented by these hallmark gene sets in both subtypes. However, fold changes resulting from the comparison of classical against basal-like PDAC samples revealed different manifestations of the respective enrichments. For example, genes involved in the epithelial-to-mesenchymal transition tended to be upregulated in both PDAC subtypes, but to a greater extent in the basal-like tumors. The same trend was observed for genes known to be upregulated upon KRAS signaling, whereas the gene set containing genes known to be downregulated upon the KRAS activation showed negative enrichment scores for both subtypes, but this trend in downregulation was more pronounced in the basal-like subtype. For gene sets related to interferon and inflammation response, positive NES values were determined in both subtypes. However, genes comprised in these sets again tended to be upregulated more strongly in the basal-like subtype. Finally, positive NES values for the glycolysis gene set and negative NES observed for oxidative phosphorylation indicated a stronger manifestation of the Warburg effect in the basal-like subtype [27]. Interestingly, genes known to be upregulated in pancreatic beta cells (gene set PANCREAS_BETA_CELLS) tended to be downregulated in both subtypes, but again this was pronounced in the basal-like subtype. In sum, these findings support the view that worse disease outcome observed for basal-like PDAC is consistently associated with pronounced dedifferentiation, indicated by impaired exocrine and endocrine functions as well as overall enhanced deregulation of cancer hallmark pathways.

### 2.3. Transcriptional Regulation of RBPs in PDAC

Aiming to characterize deregulated RBP expression in PDAC subtypes, a total number of 1542 RBPs described by the RBP census from Gerstberger et al. [28] was considered. RNA expression data for the majority of these (1499) was contained in the respective transcriptome data. A total of 290 RBPs were determined differentially expressed in PDAC compared to nonmalignant tissue, using the aforementioned criteria (130 up, 160 down; Supplementary Table S2). The comparison between nonmalignant pancreatic tissue and the classical subtype yielded 125 upregulated RBPs and 171 downregulated RBPs. In the basal-like subtype, upregulation was observed for 128 RBPs, whereas 193 were decreased. To link deregulated expression with prognostic relevance, survival analyses based on RNA expression levels in the TCGA PDAC cohort were performed. Applying a rather moderate significance threshold (log-rank test *p*-value < 0.25), we could identify 44 RBPs that were upregulated in the complete PDAC cohort or at least in one of the subtypes and furthermore showed an unfavorable prognostic value, i.e., the hazard ratio (HR) between high and low expression was greater than one. We will refer to these RBPs as PDAC onco-RBPs (PoRs) in the following (Supplementary Table S3). Analogously, we determined 104 RBPs downregulated in the PDAC samples that showed better survival probabilities (HR<1) when expressed at a higher level, which we will refer to as PDAC tumor suppressor-RBPs (PsRs; Supplementary Table S4). In accordance to the general trend observed for aberrant gene expression in PDAC, deregulation of RBP expression followed the same tendencies in both subtypes. Only DDX53, one of the PoRs showing upregulation in the basal-like subtype and associated with an adverse prognostic ($log_{2}FC=2.9$; HR=2.1), was significantly downregulated in the classical subtype ($log_{2}FC=-1.2$; HR=0.4). However, it has to be mentioned that the base expression of this RBP was considerably low (average CPM = 0.005 in pancreas).

**Figure 3:**
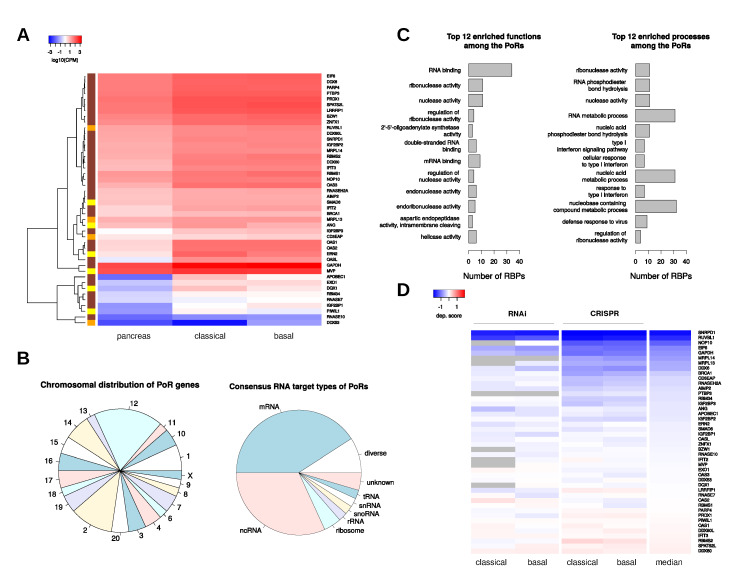
Properties of PDAC onco-RBPs (PoRs). (**A**) Average normalized RNA expression values (log10 CPM) of the 44 PoRs in pancreas as well as in classical and in basal-like PDAC samples. The color bar on the left side encodes subtype specificity of oncogene properties (yellow—upregulated and adverse prognostic only in classical PDAC; orange—upregulated and adverse prognostic only in basal-like PDAC; brown—upregulated and adverse prognostic in both PDAC subtypes). (**B**) Chromosomal distribution (**left**) and consensus RNA target types (**right**) of the PoRs. (**C**) Top 12 significantly (FDR < 0.05) enriched functions (left) and biological processes (right) among the PoRs. Gene Ontology terms are sorted according to the significance of their enrichment. (**D**) Average dependency scores of the PoRs in PDAC subtype specific cell lines obtained by knockdown via RNAi or CRISPR knockout. Gray color denotes missing values.

For this study, we focused on the PoRs (Figure 3A), since these represent factors that may promote dedifferentiation and diseases progression and may thus present therapeutic targets eligible for treatment by inhibition, as proposed for LIN28B [29] and other RBPs. Among the 44 PoRs were proteins previously implicated in PDAC progression, e.g., IGF2BP1-3 [13,14,15,16,17], IFIT3 [30], EXO1 [21], or PTBP3 [23]. However, there were also proteins that, to the best of our knowledge, have not yet been associated with oncogenic potential in pancreatic cancer, e.g., OAS proteins, RBM34 or DQX1. Remarkably, some protein families seemed to be enriched among the PoRs. The complete IGF2BP and OAS families of RBPs were included, as well as four members of the DEAD-box helicase family, two members of the IFIT family, and three RNase subunit coding genes. The strongest upregulation in PDAC samples in terms of fold change was observed for the RNA-editing enzyme APOBEC1 ($log_{2}FC=8.02$) followed by OASL, DQX1, and ERN2. All of these showed $log_{2}$ fold changes in mRNA expression above five, i.e., had average expression levels more than 32-fold higher in PDAC samples compared to normal pancreatic tissues. Comparisons regarding the expression of the PoRs in PDAC subtypes revealed that all three members of the IGF2BP family showed pronounced upregulation in the basal-like subtype. However, only for IGF2BP1 significantly higher abundance was observed in the basal-like subtype when comparing both subtypes directly ($log_{2}FC=1.01$; FDR=0.01). Notably, IGF2BP1 also showed a distinctly stronger upregulation in each subtype than the other two IGF2BPs, supporting reports on its pivotal oncogenic potential [31]. The loss of SIRT6 in PDAC tumor models was associated with upregulation of LIN28B and consequently increased expression of the let-7 targets HMGA2, IGF2BP1, and IGF2BP3 [18]. In agreement, these let-7 targets as well as LIN28B were enhanced in PDAC samples. However, instead of downregulation, this was accompanied with a modest upregulation of SIRT6 in TCGA PDAC samples ($log_{2}FC=0.4$). This suggests that other additional mechanism underlie upregulation of LIN28B in PDAC leading to enhanced expression of oncogenic factors including IGF2BP1 and HMGA2, as previously reported [20]. Besides IGF2BP1, DDX53 also showed a significantly pronounced upregulation in the basal-like subtype. The enhanced expression of both RBPs was associated with significantly reduced survival probability in the basal-like subtype whereas hazard ratios below one were observed in the classical subtype for both RBPs. In contrast, ANG, APOBEC1, DQX1, ERN2, and PIWIL1 were significantly upregulated in the classical compared to the basal-like subtype samples (Figure 3A). Furthermore, these five RBPs showed hazard ratios above one associated with significant differences in the survival probabilities only in the classical PDAC samples. The same trends in expressional differences between the tumor subtypes could be recapitulated in PDAC cell lines, classified as being classical or basal-like origin, as determined by Yu et al. [32], for IGF2BP1, ANG, APOBEC1, and ERN2 (Supplementary Figure S2A, Supplementary Table S5).

### 2.4. Characterization and Interactions of the PoRs

The chromosomal distribution of the PoR genes revealed a conspicuous enrichment of chromosome 12, since eight of these 44 RBPs (18%) are located on this chromosome (Figure 3B). This is considerably more than expected, since only around six percent of all RBPs are located on chromosome 12. Similar numbers (≈5%) are obtained when considering the fraction of all protein-coding genes located on chromosome 12 as well as those protein-coding genes that were differentially expressed in PDAC and that are located on this chromosome (Supplementary Figure S2C). The PoRs located on chromosome 12 are OAS1-3, OASL, APOBEC1, GAPDH, RBMS2, and PIWIL1. The inspection of consensus target RNA types, as reported in the RBP census, revealed a four-fold bigger fraction of the PoRs binding to unspecified noncoding RNAs when compared to all RBPs (Figure 3B, Supplementary Figure S2B). Among these RBPs were the viral RNA binding proteins OAS1-3, OASL, IFIT2, and IFIT3, as well as the vaultRNA (vtRNA) binding protein MVP.

Functional enrichment analyses using the 44 PoRs revealed that, besides the obvious enrichment of RNA-binding functionality, proteins influencing nuclease activity were significantly enriched among those RBPs (Figure 3C, Supplementary Table S6). This category contained the nucleases RNASE7, RNASEH2A, RNASE10, and EXO1, as well as proteins regulating nucleases, like the OAS-protein family that are known to activate RNase L activity upon viral infection [33]. Helicase activity was also significantly enriched among PoRs, since they comprised four DEAD-box helicases (DDX53, DDX6, DDX60, DDX60L) as well as RUVBL1 and DQX1, both described to at least possess DNA-helicase activity. In addition, with ZNFX1, a further reported helicase was included in the PoR list, although not yet annotated as such in Gene Ontology [34]. Interestingly, analysis of enriched processes revealed that a substantial number (9/44) of PoRs play a role in the viral defense response (Figure 3C, Supplementary Table S6). These include IFTI2 and IFIT3, OAS1-3 and OASL, APOBEC1, DDX60, and MRPL13. In addition, two further helicases among the PoRs, DDX60L and ZNFX1, were recently described to be involved in the human innate immune response against viral infection [35,36].

To evaluate the “essentiality” of PoRs in cancer cells, we queried dependency scores reported by the DepMap project for PDAC-derived cell lines (Figure 3D, Supplementary Table S7), classified as classic- or basal-like. RNAi-mediated depletion as well as CRISPR-mediated deletion data of the respective RBPs were extracted from the DepMap data [37,38]. In general, dependency scores reported by DepMap are supposed to indicate how essential a specific gene is for cell survival and propagation in 2D cell culture. Dependency scores below –1, the median dependency score of all pan-essential genes, are considered to indicate core essential genes. In the RNAi screens, although largely negative, most of the PoRs showed average dependency scores close to zero, implying only minor proliferation effects when depleted (Figure 3D, Supplementary Table S7). In general, dependency scores of the PoRs obtained from the CRISPR screens were lower compared to those from the RNAi screens. As expected, the lowest dependency scores were observed for the basal factors of ubiquitous function, for instance SNRPD1, a crucial component of the SMN-Sm complex mediating spliceosomal snRNP assembly. Notably however, in general, determined dependency did not reflect the impact of the PoRs as determined prior by hazard ratios or upregulation in PDAC or any of the two PDAC subtypes. This suggests that in-vitro-determined dependency scores are only of minor value for evaluating the oncogenic potential of PoRs.

**Figure 4:**
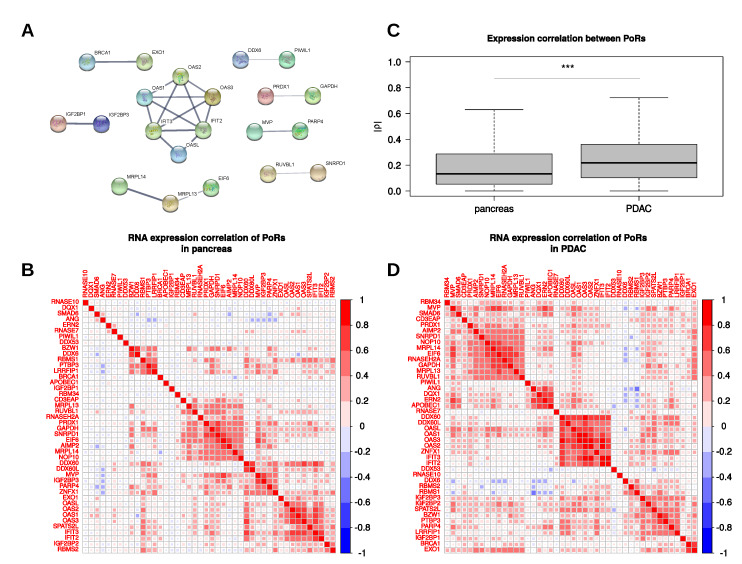
PoR interactions. (**A**) Known physical interactions between PoRs according to the STRING database. (**B**,**D**) Spearman’s correlation coefficients of the PoR RNA expression in pancreas (**B**) and PDAC (**D**) RNA-seq samples. (**C**) Distribution of Spearman’s correlation coefficient magnitudes (|ρ|) obtained from comparisons among the 44 PoRs in pancreas and PDAC RNA-seq samples. ***: Mann–Whitney test *p*-value < 0.001.

To investigate whether the 44 PoRs might act together via common protein complexes or at least influence each other, we searched for known protein–protein interactions (PPI) and analyzed relations of their RNA expressions in PDAC. The search for known PPI using the STRING-database (string-db.org, [39]) revealed a highly connected network consisting of the four OAS proteins as well as the two members of the IFIT family IFIT2 and IFIT3 (Figure 4A). Furthermore, the mRNAs encoding the respective proteins showed highly correlated RNA expression values in pancreatic tissues. These correlations were even more pronounced in the PDAC samples, where a distinct cluster of proteins with very high expression correlation was formed (Figure 4B,D). Besides the OAS and IFIT proteins, this cluster contained the helicases DDX60, DDX60L, and ZNFX1, suggesting a collective upregulation of RBPs exerting antiviral functions in PDAC. The high degree of expression correlation of these RBPs, here termed the OAS-IFIT-cluster, could also be recapitulated when considering the PDAC subtypes alone (Supplementary Figure S3A,B), suggesting subtype independent function. Another PPI reported by STRING was found between EXO1 and BRCA1. This interaction was speculated to augment the recruitment of EXO1 to DNA double-strand breaks, especially during the G2 phase of the cell cycle [40]. The Spearman correlation coefficient (ρ) of the RNA expression of these two RBPs increased from 0.16 in pancreas to 0.61 in PDAC, suggesting a stronger association of them in the tumors. Whereas a strong and discrete association cluster of both factors was observed in the classical subtype, the correlation was weaker and the cluster appeared less discrete in the basal-like subtype (Supplementary Figure S3A,B). Another example of a reported PPI that was pronounced stronger by expression correlation in PDAC than in normal pancreas is IGF2BP1 und IGF2BP3. Here, Spearman’s ρ increased from −0.04 to 0.43. However, although the overall expression correlation coefficients between IGF2BP1 and the other PoRs tended to increase in the PDAC samples, besides IGF2BP3 only SMAD6 and EXO1 showed a moderate positive correlation with IGF2BP1 (ρ=0.34, each), whereas the correlation coefficients between IGF2BP1 and the remaining PoRs were considerably below 0.3. This suggested that IGF2BP1 exerts its roles largely independent of other PoRs in PDAC as well as the subtypes. In contrast, the other two IGF2BP members showed high correlation (ρ > 0.3) to several other PoRs in the PDAC samples, for example to each other, LRRFIP1, SPATS2L, and to the abovementioned cluster of PoRs with antiviral functions. These correlations were considerably lower in nonmalignant pancreas samples, supporting the oncofetal expression pattern of IGF2BPs [41]. In general, a trend for stronger correlations (positive as well as negative) between PoRs could be observed in PDAC compared to normal pancreas (Figure 4C). Despite the conserved association of the OAS-IFIT-cluster in PDAC subtypes, most association clusters showed variable extent and composition between both subtypes.

### 2.5. Post-Transcriptional Regulation of the PoRs by microRNAs

**Figure 5:**
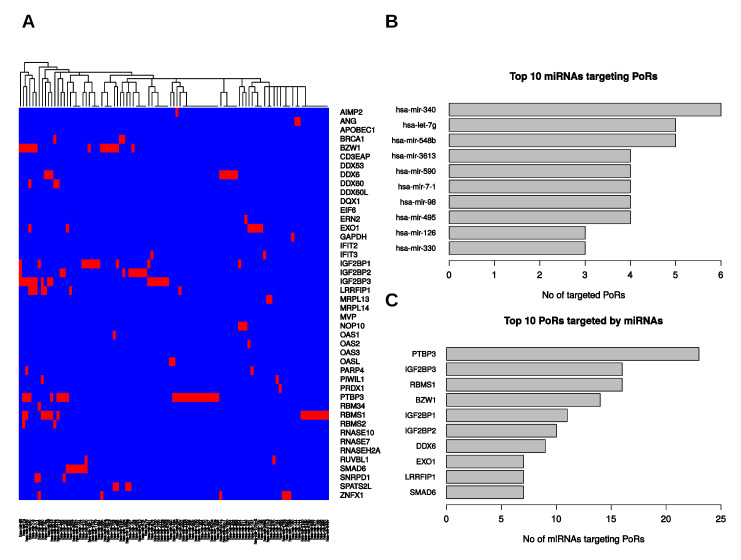
Putative interactions between PoRs and microRNAs. (**A**) Predicted PoR–miRNA-interactions that were associated with significant (*p* < 0.05) negative expression correlation (Spearman). Red color encodes inferred interaction, blue means no interaction. (**B**) Top 10 miRNAs targeting the most PoRs. (**C**) Top 10 PoRs targeted by the most miRNAs.

MicroRNAs (miRNAs) and RBPs form regulatory networks modulating mRNA expression. One prominent example in cancer is the LIN28B/IGF2BP-containing network, which antagonizes tumor-suppressive regulation by let-7 miRNAs. Whereas LIN28B interferes with let-7 biogenesis, IGF2BPs impair targeting of let-7 on specific transcripts [20]. Aiming to reveal additional miRNAs regulating PoRs, we determined microRNAs predicted to bind to their respective mRNAs by querying eight different prediction databases. We required a certain mRNA–miRNA binding to be predicted by at least two of the eight databases to be considered further. Subsequently, we performed Spearman’s correlation tests using TCGA small RNA-seq data to filter out those predicted target interactions without significant negative correlation of expression between the respective miRNA and RBP-encoding mRNA in PDAC (ρ<0, p<0.05). Thus, we limited the prediction results to those relevant for the provided PDAC data. This resulted in 99 different miRNAs predicted to target 31 of the 44 PoRs (see Figure 5A, Supplementary Table S8). Notably, three of the obtained miRNAs (miR-126, miR-454, miR-3613) were included in a recently reported miRNA-signature consisting of seven miRNAs that are downregulated in PDAC and were proposed as prognostic markers for pancreatic cancer [11]. The RBP predicted to be bound by the highest number of different miRNAs was PTBP3 (23), followed by IGF2BP3 and RBMS1 (16 each). Further, the other two members of the IGF2BP family were also among the 10 PoRs predicted to be bound by the most miRNAs (Figure 5C), and each IGF2BP was predicted to be targeted by at least one let-7 family member. Another microRNA we determined to be a putative regulator of IGF2BP1 and IGF2BP3 expression was miR-491. Previously, this microRNA was found to suppress cell proliferation and invasion in non-small cell lung cancer by inhibiting IGF2BP1 [42]. Interestingly, miR-491 was recently reported to act as a tumor-suppressive microRNA by inhibiting the known IGF2BP targets IGF2 and HMGA2 [43,44].

### 2.6. Selection of Candidate PoRs for Therapeutic Targeting

**Figure 6:**
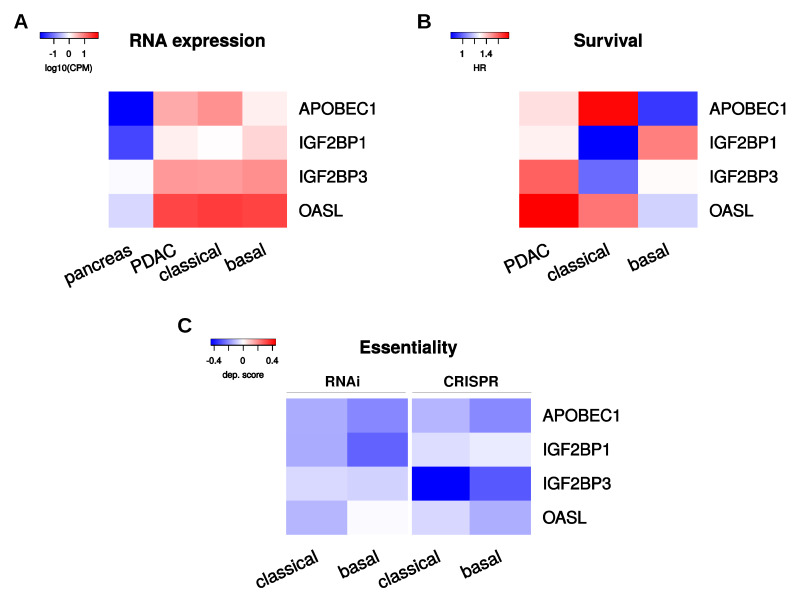
Selected PoRs. (**A**) Average normalized RNA expression values (log10 CPM) of the four selected PoRs in pancreas and PDAC samples comprised of the whole (PDAC) tumor sample set as well as subtype specific subsets. (**B**) Hazard ratios (HR) of the four selected PoRs determined from overall survival rates between low and high RNA expression. (**C**) Average dependency scores of the four selected PoRs obtained from RNAi-derived depletion as well as CRISPR-derived deletion in classical and basal-like PDAC derived cell lines.

Aiming to select RNA binding proteins that could serve as promising candidates for therapeutic targeting, we evaluated PoRs based on two criteria. First, we picked PoRs with substantial upregulation or de novo synthesis in PDAC, as indicated by very low average RNA expression in healthy pancreas tissues (<1 CPM) and at least six-fold upregulation in one of the PDAC subtypes. Moreover, we required the candidates to have negative average dependency scores upon RNAi as well as upon CRISPR loss-of-function studies in classical and basal-like PDAC cell lines, indicating a necessity for proliferation and tumor cell survival, at least in vitro. Using these filter criteria, four RBPs were unveiled—namely, APOBEC1, IGF2BP1, IGF2BP3, and OASL (Figure 6). APOBEC1 was the PoR with the strongest relative upregulation in PDAC, compared to nonmalignant pancreatic tissue, and with substantially enhanced expression in the classical compared to the basal-like subtype (Figure 6A). Furthermore, high expression of APOBEC1 was associated with adverse disease outcome only in the classical subtype (Figure 6B, Supplementary Figures S4A and S5A,B). This suggests APOBEC1 as a prime candidate target PoR in the classical subtype. IGF2BP3 and OASL showed only modest variability in upregulation between the PDAC subtypes (Figure 6A) and similar or insignificant survival probabilities in the subtypes (Figure 6B, Supplementary Figures S4C,D and S5E–H). This may indicate that these two PoRs serve largely subtype-independent tumor-promoting roles, suggesting them as subtype-independent candidate targets. IGF2BP1 was the candidate PoR with the highest hazard ratio determined in the basal-like subtype (Figure 6B, Supplementary Figures S4B and S5C,D) and was stronger upregulated in this subtype (Figure 6A). This suggests IGF2BP1 as a major candidate target in the basal-like subtype.

## 3. Discussion

In this study we identified 44 RNA binding proteins (RBPs) salient in the context of pancreatic ductal adenocarcinomas (PDAC) due to their elevated RNA expression and adverse prognostic values. We termed these pancreatic oncogenic RBPs (PoRs). Among this small set of RBPs, several protein families were enriched, since it contained the whole IGF2BP and OAS family as well as several DEAD-box helicases and RNase genes. Some of these RBPs seem to form interaction networks with each other, as suggested by protein–protein-interaction data and further supported by the high correlation of RNA expression among them. We identified a network of coexpressed RBPs that are known to be part of the innate immune response against viral infections. This network was formed by OAS1-3, OASL, IFIT2, and IFIT3, as well as the helicases DDX60, DDX60L, and ZNFX1. Whether the upregulation of these RBPs is due to infections accompanying the progression of at least a subset of the investigated pancreatic tumors, due to a common pathway deregulated upon infection and tumor development (e.g., inflammation related), or is just due to coincidence still has to be investigated. However, our network analyses suggest some RBPs, like IGF2BP1, to act rather independently from the other identified PoRs. Incorporating small RNA-seq data and miRNA predictions, we observed that most of the 44 selected RBPs are putative targets of only a few miRNAs commonly downregulated in PDAC.

We further focused on RBPs upregulated in either one or both considered PDAC subtypes, showing consistently negative dependency scores and hazard ratios greater than one in PDAC and/or subtypes. These proteins might be particularly suitable for therapeutic inhibition treatments since, due to low or absent expression in the “normal” pancreas, they likely represent proteins important for the cancer but dispensable for healthy pancreas cells. Prime candidates fitting these criteria are APOBEC1, OASL, IGF2BP1, and IGF2BP3. APOBEC proteins are enzymes capable of introducing RNA and DNA modifications and able to restrict viral infections by catalyzing mutations in viral genomes, but are also thought to drive tumor evolution by introducing somatic mutations [45,46]. APOBEC1 was first connected to cancer when transgenic mice and rabbits expressing the protein in their livers developed liver cancer [47]. Tumorigenesis was attributed to editing and consequently repression of the mRNA of NAT1/EIF4G2 [48]. However, we observed a slight upregulation of the EIF4G2 gene in PDAC ($log_{2}FC=0.56$). Furthermore, we found A1CF, the APOBEC1 complementation factor essential for the mRNA editing functionality of APOBEC1 [49], to be significantly downregulated in PDAC samples compared to normal pancreas. However, in a recent study of 32 tumor types from the TCGA, the authors found a striking correlation of APOBEC1 upregulation in tumors bearing a high number of in-frame indel mutations in various cancer cohorts including PDAC. In addition, A1CF was not found to be differentially expressed in the samples with a high number of mutations compared to samples showing low mutation levels [50]. This suggests that the oncogenic potential of APOBEC1 in PDAC is based on its DNA-editing capability rather than on its RNA-modifying function. OASL, a member of the OAS protein family, like APOBEC1, is associated to the innate immune defense against viral infections. In contrast to OAS1-3, human OASL is lacking the 2′-5′ oligoadenylate synthase activity, responsible for the name of the protein family. OASL has been reported to exert antiviral as well as proviral function, depending on various mechanisms and phase of infection [33]. Lv et al. recently reported that inhibition of OASL in lung-cancer-derived cells inhibits their proliferation [51]. However, the mechanism causative for the pro-proliferative effect of OASL still has to be elucidated. IGF2BP1 and IGF2BP3 are *bona fide* oncofetal proteins upregulated in a variety of malignancies and were shown to enhance the expression of genes related to cell migration and proliferation [41,52,53]. In previous studies, we could show that IGF2BP1 stabilizes its RNA targets by antagonizing miRNA-impaired gene expression in different cancer models [20,31]. In particular, we could show that IGF2BP1 shields the transcripts of LIN28B and HMGA2 as well as its own mRNA from let-7-mediated downregulation in ovarian cancer-derived cells, thus promoting an aggressive tumor phenotype [20]. A similar mechanism of a self-promoting network including IGF2BP1 and IGF2BP3 may be active in PDAC, preventing miR-491 targets to be suppressed. We found this miRNA to likely be a putative regulator of both IGF2BPs and it has already been published that miR-491 suppresses IGF2BP1 as well as the known IGF2BP targets IGF2 and HMGA2 [42,43,44]. Furthermore, it was demonstrated that the association of IGF2BPs and their target mRNAs is mediated by N6-methyladenosine (m${}^{6}$A) modifications [54,55]. Although we did not investigate m${}^{6}$A-modifications in PDAC for the present study, it is tempting to speculate that the oncogenic potentials of IGF2BP1 and 3 in PDAC also depend on this modification. However, this still has to be elucidated. Notably, small molecule or nucleic acid-derived inhibitors for our prime candidates or their homologues have already been reported. Inhibition by APOBEC3 proteins has been addressed in the context of HIV infection and cancer [46], suggesting that inhibition of APOBEC1 is feasible. The small molecule BTYNB was reported to inhibit IGF2BP1 from binding to c-MYC mRNA and showed moderate cytotoxic effects in various cancer-derived cells [56]. We recently demonstrated, that BTYNB impairs IGF2BP1-driven cell cycle progression and tumor growth in murine ovarian cancer models (Müller et al., in revision). A recent in silico screening for inhibitors of OAS1-3 yielded several compounds able to inhibit ATP binding of these RBPs, which should result in an impaired synthase activity [57]. Furthermore, divalent metal ions like zinc ions were also shown to inhibit the enzymatic activity of OAS1 and OAS2 [58]. Notably, a recently conducted meta-analysis of different studies concluded that high dietary zinc uptake can significantly reduce the risk for developing pancreatic cancer [59].

In conclusion, our studies identify various oncogenic RBP candidates in pancreatic cancer. Some of these proteins have been reported as tumor-promoting factors in various experimental cancer models. Nonetheless, further experimental validation of proposed oncogenesis-driving roles of identified candidate PoRs in pancreatic tumor models is required, since the data presented here solely rely on publicly available high-content studies. Importantly however, for some of the candidate proteins, therapeutic targeting strategies have already been reported or appear feasible.

## 4. Materials and Methods

### 4.1. RNA-seq Data Processing, Differential Expression, and Survival Analysis

We obtained gene-level RNA-seq read counts of TCGA primary tumor PDAC samples and GTEx V7 normal pancreas tissue via the GDC data portal (portal.gdc.cancer.gov) and the GTEx portal (gtexportal.org), respectively. By combining these data, we got read count information of 53045 genes for 177 primary tumor samples and 248 normal pancreas tissue samples. Differential gene expression was assessed using R/edgeR v3.28.0 [60] by applying trimmed mean of M values (TMM) normalization. CPM transformation was utilized to obtain normalized expression values. False discovery rate (FDR) values below 0.05 and at least two-fold changes in mean expression ($|log_{2}FC|\geq 1$) were considered as thresholds for the determination of differential gene expression. MiRNA expression data were also obtained by downloading read count data of TCGA PDAC samples via the GDC data portal. CPM values were generated after applying TMM normalization using edgeR. Survival analyses were performed using normalized TCGA RNA expression data processed as mentioned above and associated clinical data obtained from the GDC data portal. The log-rank test was implemented in an R-script according to the description in [61]. High and low expression groups were separated by the respective gene’s median RNA expression value.

### 4.2. Functional Enrichment Analyses

Gene set enrichment analyses were performed using the GSEA v3.0 software [25] and MSigDB v7.0 gene sets [26], applying the pre-ranked test, 1000 permutations, and the classical scoring scheme. Gene annotation enrichment analyses were performed using Cytoscape v3.7.0 [62] and the ClueGO plugin v2.2.5 [63]. For analysis of enriched molecular functions using the Gene Ontology release 2020-01-28 [64,65], we applied the right-sided hypergeometric test, a cutoff-value for Benjamini–Hochberg corrected *p*-values of 0.05, and a minimum GO-level of four.

### 4.3. RNA Expression and Dependency Scores in PDAC Cell Lines

RNA expression values (TPM) and dependency scores were obtained from the DepMap project [37] via the R-package depmap v1.0.0 using the 19Q3 release. Classification of PDAC cell lines into basal-like or classical subtype origin were obtained from the supplementary material of [32]. Only cell lines associated to a subtype class with an FDR less than 0.05 were considered.

### 4.4. Protein–Protein Interaction Analysis

In order to get protein–protein interactions, we queried the STRING v11 database [39] via the STRING website (string-db.org). To obtain only known interactions, we restricted the search to information experimentally determined or from curated databases, applying an interaction score of 0.4.

### 4.5. MicroRNA Binding Prediction

To infer putative RBP-miRNA bindings, we first queried for predicted miRNA bindings utilizing the R-package multiMiR v1.8.0; Database Version 2.3.0 [66] using all eight prediction databases and the default prediction cutoff of 20%. We removed all predicted bindings predicted by less than two different databases. From the remaining predictions, we chose those as putative interactions that showed a significant negative Spearman correlation (ρ<0; *p*-value <0.05) in their expression values to the RNA expression of the respective RBP. For this purpose, we compared RBP CPM values obtained as described above with TCGA miRNA data obtained from the same tumor samples.

## Supplementary Materials

The following are available online at https://www.mdpi.com/1422-0067/21/11/4190/s1, supplementary_figures.pdf: supplementary figures, Table S1: GSEA DGE-Pancreas-PDAC curated gene sets, Table S2: Differential gene expression and Survival analyses of RBPs, Table S3: Differential gene expression and Survival analyses of PoRs, Table S4: Differential gene expression and Survival analyses of PsRs, Table S5: Gene expression of RBPs in PDAC cell lines, Table S6: Significantly enriched molecular functions and biological processes among the PoRs, Table S7: Dependency scores of PoRs in PDAC-derived cell lines, Table S8: Predicted PoR-miRNA binding.
