# Supplementary material for: RNA Binding Proteins as Drivers and Therapeutic Target Candidates in Pancreatic Ductal Adenocarcinoma
Source: Int J Mol Sci. 2020 Jun 11;21(11):4190. doi: 10.3390/ijms21114190 (PMC7312628; doi:10.3390/ijms21114190)
Supplement: Supplementary file 1 [file ijms-21-04190-s001.zip › supplementary_figures.pdf]

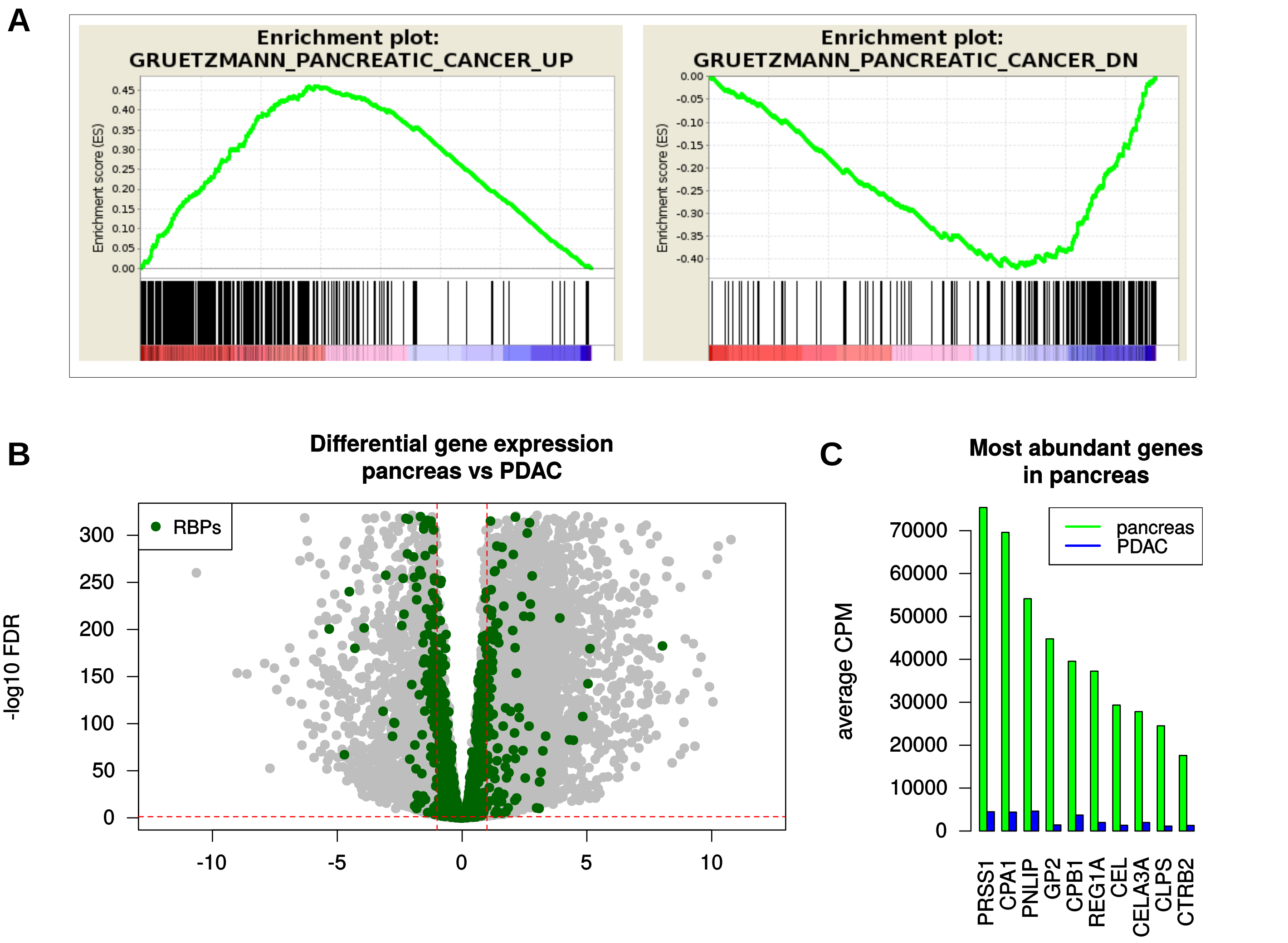

Supplementary Figure 1: Comparison of GTEx pancreas and TCGA PDAC RNA-seq samples. (A) Exemplary gene set enrichment analysis (GSEA) plot of selected gene sets related to pancreatic cancer. (B) Volcano plot showing the distribution of log2 fold changes and log10 FDR values resulting from the analysis of differential expression of protein coding genes. Green points mark RNA binding proteins (RBPs). (C) Average RNA expression values (CPM) from pancreas (green) and PDAC (blue) samples of the 10 genes with the highest gene expression in pancreas.

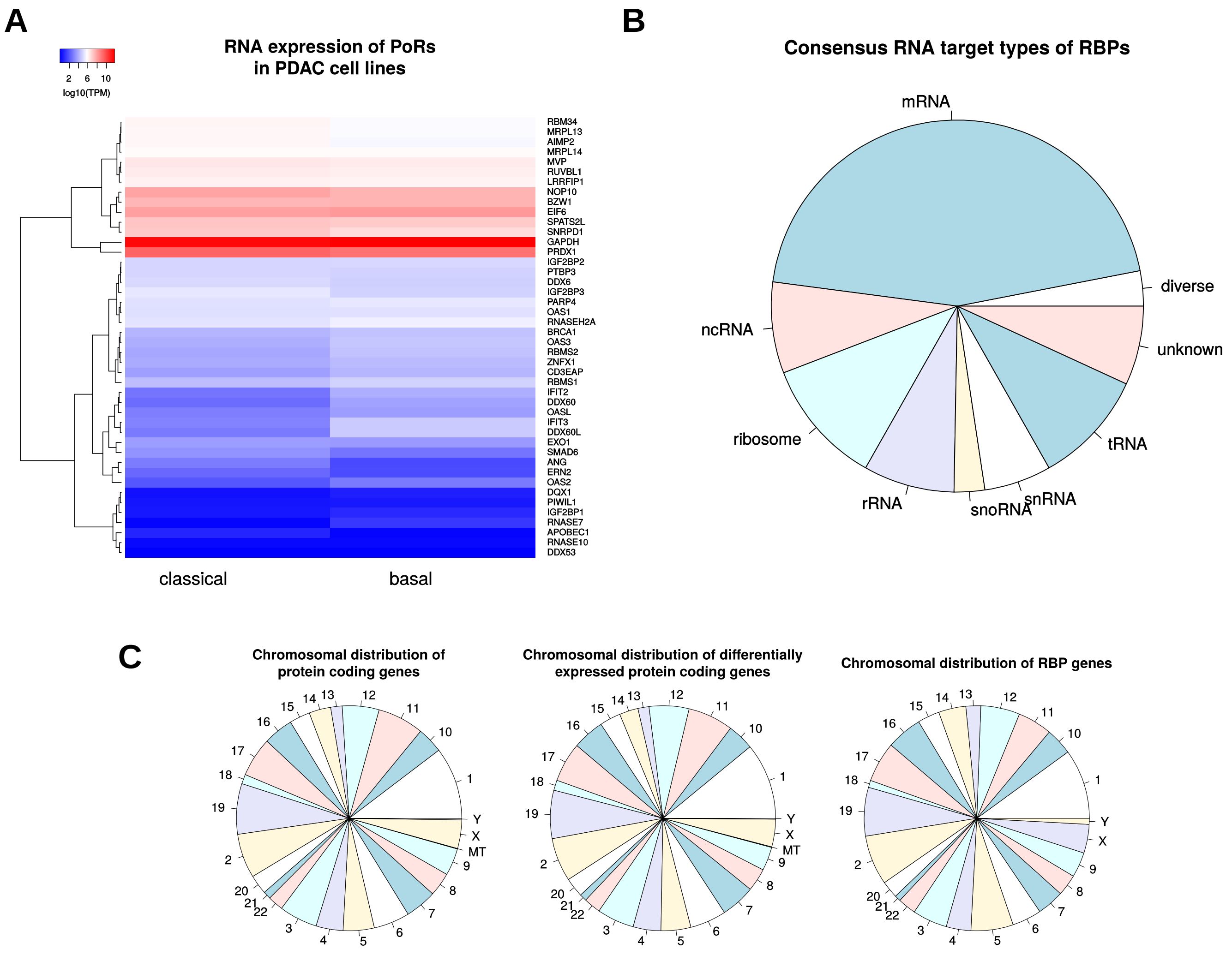

Supplementary Figure 2: Properties of PoRs. (A) Heatmap showing the average normalized RNA expression values (log10 TPM) of the 44 PoRs in classical and basal-like PDAC-derived cell lines. (B) Consensus RNA target types of RBPs. (C) Chromosomal distribution of all protein coding genes (left), protein coding genes differentially expressed between pancreas and PDAC (middle) and RBPs (right).

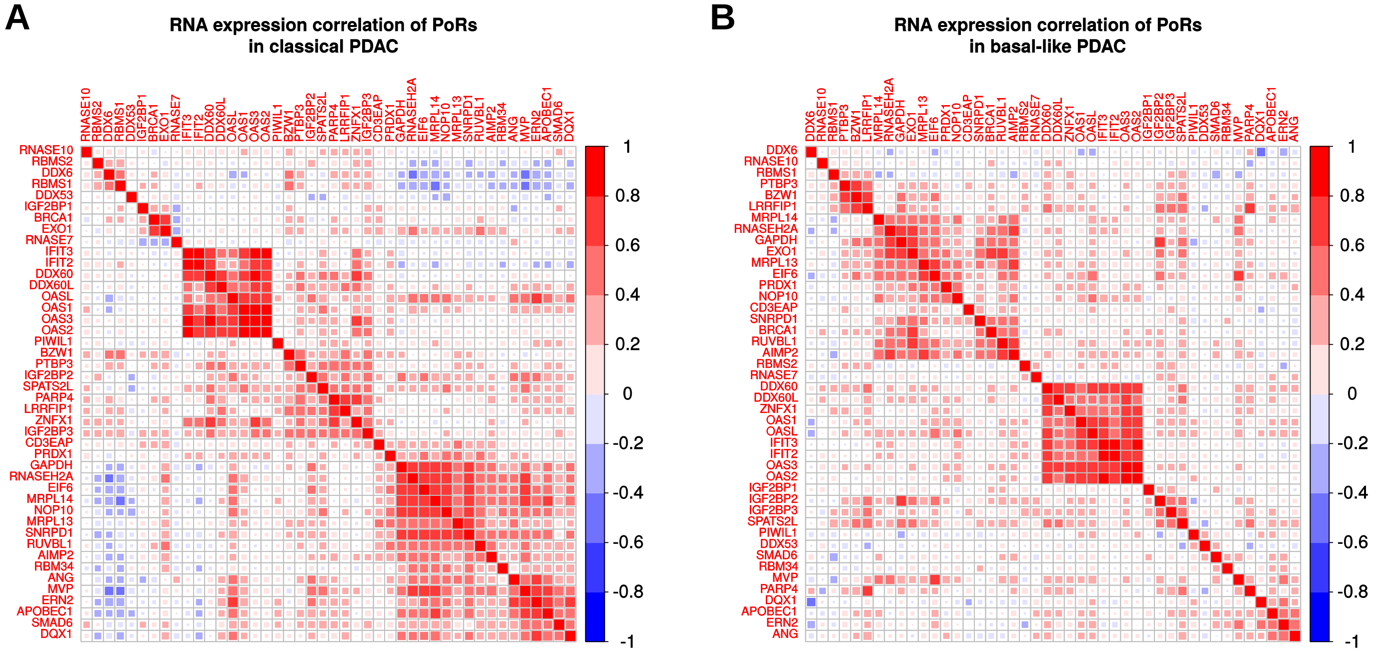

Supplementary Figure 3: Spearman correlation coefficients (ρ) of PoR RNA expression in classical (A) and basal-like (B) PDAC RNA-seq samples.

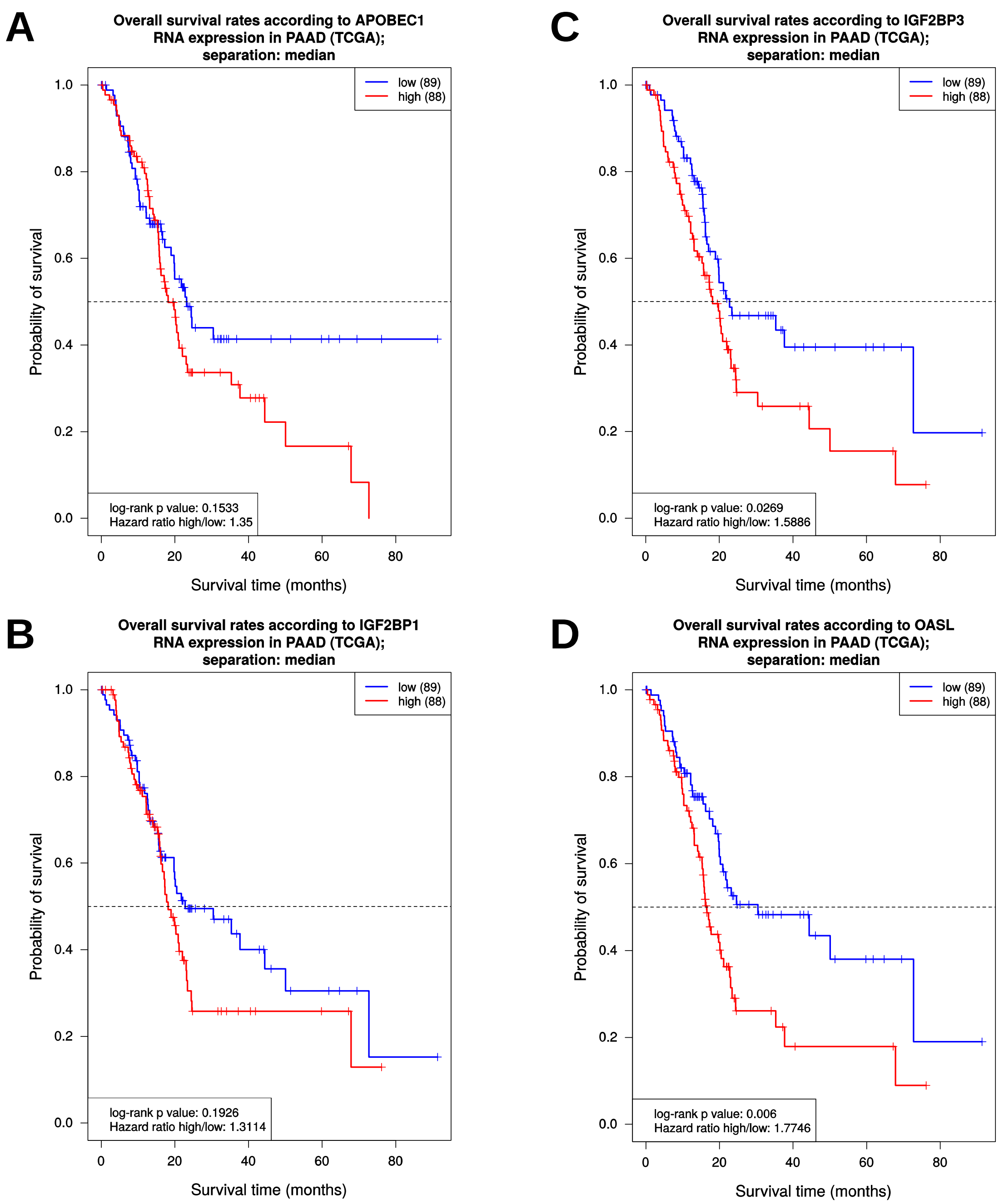

Supplementary Figure 4: Kaplan-Meier-curves showing overall survival rates for low (blue) and high (red) RNA expression in PDAC. (A) APOBEC1, (B) IGF2BP1, (C) IGF2BP3 and (D) OASL.

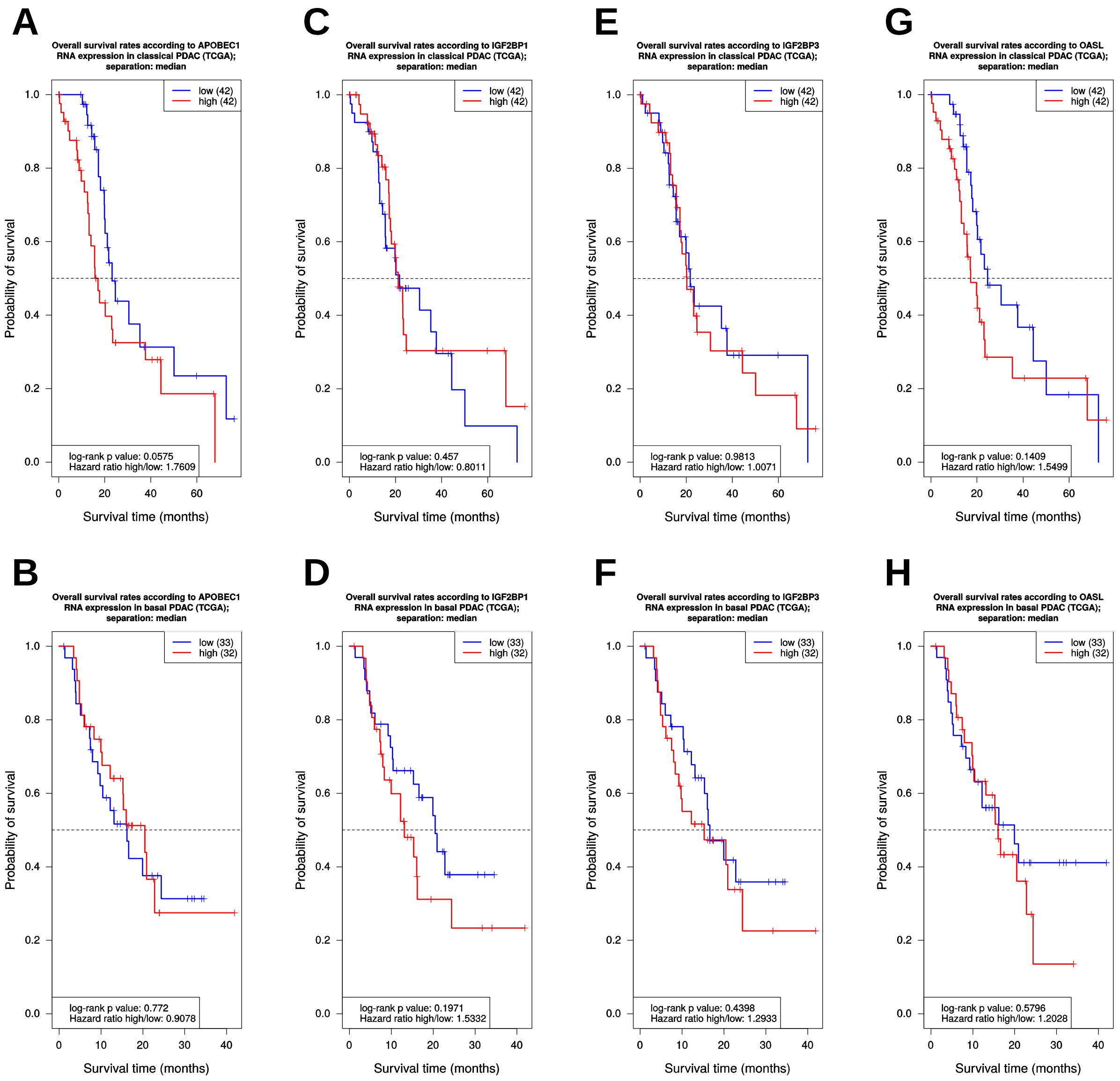

Supplementary Figure 5: Kaplan-Meier-curves showing overall survival rates for low (blue) and high (red) RNA expression in PDAC subtypes. Upper panel shows classical subtype, lower panel shows basal-like subtype. (A, B) APOBEC1, (C, D) IGF2BP1, (E, F) IGF2BP3 and (G, H) OASL.
